# Obesity Does Not Increase the Risk of Asthma Readmissions

**DOI:** 10.3390/jcm9010221

**Published:** 2020-01-14

**Authors:** Francisco-Javier Gonzalez-Barcala, Juan-José Nieto-Fontarigo, Tamara Lourido-Cebreiro, Carlota Rodríguez-García, Maria-Esther San-Jose, Jose-Martín Carreira, Uxio Calvo-Alvarez, Maria-Jesus Cruz, David Facal, Maria-Teresa Garcia-Sanz, Luis Valdes-Cuadrado, Francisco-Javier Salgado

**Affiliations:** 1 Department of Medicine, Universidade de Santiago de Compostela, 15705 Santiago de Compostela, Spain; Francisco.javier.gonzalez.barcala@sergas.es (F.-J.G.-B.); Luis.Valdes.Cuadrado@sergas.es (L.V.-C.); 2 Spanish Biomedical Research Networking Centre-CIBERES, 28029 Madrid, Spain; mj.cruz@vhir.org; 3 Department of Respiratory Medicine, University Hospital of Santiago de Compostela (CHUS), 15706 Santiago de Compostela, Spain; 4 Health Research Institute of Santiago de Compostela (IDIS), 15706 Santiago de Compostela, Spain; 5 Department of Biochemistry and Molecular Biology, Faculty of Biology-Biological Research Centre (CIBUS), Universidade de Santiago de Compostela, 15706 Santiago de Compostela, Spain; franciscojavier.salgado@usc.es; 6 Department of Experimental Medical Science, Respiratory Immunopharmacology group, Lund University, 223 62 Lund, Sweden; 7 Clinical Analysis Service, University Hospital of Santiago de Compostela (CHUS), 15706 Santiago de Compostela, Spain; sanjosec@yahoo.com; 8 Department of Radiology, Universidade de Santiago de Compostela, 15706 Santiago de Compostela, Spain; josemartin.carreira@usc.es; 9 Department of Respiratory Medicine, University Hospital of Ferrol, 15045 Ferrol, Spain; uxve@msn.com; 10 Department of Respiratory Medicine, Hospital Vall d’Hebron, 08023 Barcelona, Spain; 11 Department of Developmental Psychology, Universidade de Santiago de Compostela, 15706 Santiago de Compostela, Spain; 12 Emergency Department, Hospital O Salnes, 36600 Vilagarcía de Arousa, Spain; maytegsanz@gmail.com

**Keywords:** asthma exacerbations, obesity, hospital readmissions, prognosis

## Abstract

The relationship between obesity and asthma exacerbations is still under debate. The aim of our work is to analyse the relationship between obesity and hospital re-admissions in asthmatics. A review was retrospectively performed on all hospital admissions of adult patients due to asthma exacerbation occurring in our hospital for 11 years. All those cases with asthma as the first diagnosis in the discharge report were included, or those with asthma as the second diagnosis provided when the first diagnosis was respiratory infection or respiratory failure. Only the first hospital admission of each patient was included in this study. The Odds Ratios of a higher incidence of early/late readmissions due to asthma exacerbation were calculated using a binary logistic regression, using the body mass index (BMI) as independent variable, adjusted for all the variables included in the study. The study included 809 patients with a mean age of 55.6 years, and 65.2% were female. The majority (71.4%) were obese or overweight. No significant relationship was observed in the univariate or multivariate analyses between overweight or obesity and the early or late hospital readmissions due to asthma. Therefore, obesity does not seem to be a determining factor in the risk of asthma exacerbations.

## 1. Introduction

Obesity is a significant health problem that affects more than 500 million people in the world, and more than 1.5 billion people are overweight [1]. Asthma is also an illness with an elevated prevalence. In 2016, the Global Burden of Disease (GBD) study estimated that there were 339.4 million people worldwide affected by asthma. This represents a 3.6% increase in age-standardised prevalence since 2006 [2]. This increase in the prevalence of asthma in such a short time is probably more related to environmental than genetic factors [3,4]. It also has a large impact on the population as asthma is ranked 16th overall among the leading causes of years lived with disability, and 28th among the leading causes of burden of disease [2]. Mortality appears to be reducing, although with the significant differences between countries and age groups, it continues to be elevated. In 2016, the GBD collaboration estimated that 420,000 people in the world died from asthma (i.e., more than 1000 per day) [2,5].

Obesity is one of the factors that is being investigated as a possible cause of asthma or of a worse prognosis of the disease. Two asthma phenotypes associated with obesity have been identified. One with early-onset asthma, usually triggered by allergens, with eosinophilia and an elevated IgE, and aggravated by obesity. The other phenotype is usually of late onset, mainly in women, with low Th2 markers with an increase in leptin and interleukin 6 [6].

However, the relationship between obesity and asthma is not clear, with significant divergences between different authors in most of the features analysed. On analysing the prevalence of asthma, some authors showed that obesity was associated with significant increases in its prevalence [7,8,9], whilst others mentioned that obesity did not affect asthma [10,11], and in other cases that it may be associated with prevalence in some age groups, but not in others [12]. Regarding the control of the disease, it is still under debate, with some authors observing a poorer control in the obese population [13,14,15], whilst in other publications, the increase in weight does not appear to have an influence on the control of asthma [8,16,17]. On analysing the impact of obesity on the incidence of exacerbations, there are still discrepancies between authors. Some do not observe a relationship between the increase in weight and the incidence of exacerbations in some cases [17,18], but in other publications it is mentioned that exacerbations are more common with an increase in weight [15,19,20]. However, obesity does not appear to be associated with the severity of the exacerbations or in the time required to recover from them [21,22,23]. Regarding the quality of life, the negative effect of obesity appears to be clearer in this respect, with a worsening of quality of life with an increase in weight [13,15,24].

Due to these discrepancies, the aim of our study was to analyse the possible relationship of obesity with hospital readmissions in patients with asthma. 

## 2. Material and Methods

A review was retrospectively carried out on all hospital admissions of patients over 18 years old due to exacerbation of asthma occurring in our hospital between the years 2000 and 2010.

The data were gathered by two members of the research team by reviewing the clinical records. In cases where there were discrepancies in the evaluation of any data, the decision was made by consensus with the rest of the group members. Follow-up treatment of these patients was carried out after discharge by specialists in Respiratory Medicine or Internal Medicine.

All those cases with asthma as the first diagnosis in the discharge report were included, or those with asthma as the second diagnosis provided that the first diagnosis was respiratory infection or respiratory failure. Cases were excluded if the admission was due to another specific cause, such as pulmonary embolism or pneumonia. The patients were diagnosed and treated according to the decision of the treating physician. Only the first hospital admission of each patient was included in this study.

The personal characteristics of each patient were obtained from the clinical records in stable state, including age, gender, body mass index (BMI), classifying the patients into normal weight when they had a BMI less than 25 kg/m^2^, as overweight with a BMI greater than or equal to 25, and a BMI greater than or equal to 30 as obese [25]. Comorbidity was evaluated according to the Charlson index [26], and smoking classified as active smokers, ex-smokers, or never smoked. 

An analysis was performed on the asthma personal history, lung function based on the forced expiratory volume (FEV1) as a percentage of the reference value, the baseline severity of the asthma according to the Global Initiative for Asthma (GINA) 2006 criteria [27], having presented with any episode of near-fatal asthma (NFA) [22], and hospital emergency department (ED) visits or admissions due to asthma in the previous year and treatment after hospital discharge.

Previous year emergencies were stratified into three groups: one group of those who did not make any visit to the ED in the previous year, another that made one to three visits, and a last group that made four or more visits to the ED in the previous year. Previous year hospital admissions were stratified into three groups: one group of those who did not have any hospital admission in the previous year, another that had one hospital admission, and a last group that had two or more hospital admissions in the previous year.

Early readmission (ER) was defined as that which occurred in the following 15 days after hospital discharge, and late readmission (LR) occurred from 16 days after discharge [28].

These patients were followed-up during the inclusion period from 2000 to 2010. The follow-up period was extended for one year for patients including during 2010, hence every patient was monitored for at least one year.

The Review Board on Human Studies at our institution approved the protocol (Register Code: 2017/093).

### Statistical Analysis

The data were tested for normal distribution using the Kolmogorov–Smirnov test. The comparison of the categorical variables was performed using the Chi-squared test. For the comparison of the continuous variables, the *t*-student test was used.

The Odds Ratios (OR) of a higher incidence of ER and LR were calculated using a binary logistic regression, using body mass index as independent variable, adjusted for all the variables included in the study: age, gender, Charlson index, smoking habit, asthma severity, hospital admissions and emergency visits in the previous year due to asthma, and treatment after hospital discharge with combinations of inhaled corticosteroids and long acting beta-agonists (ICS-LABA), inhaled, or oral corticosteroids. 

All statistical analyses were performed using SPSS for Windows, Version 15.0 (SPSS Inc., Chicago, IL, USA).

## 3. Results

The study included 809 patients with a mean age of 55.6 years, and 65.2% were female. The majority (71.4%) were obese or overweight (Table 1). As expected, the obese patients were more likely to be female, older, with more comorbidity, less frequent smokers, higher asthma severity, and with more frequent emergency visits (Table 1). There were early readmissions by 18 patients (2.2%), late readmissions in 168 patients (20.8%), and 54 (7.8%) patients with two or more late readmissions.

No significant relationship was observed in the univariate or multivariate analyses between overweight or obesity and the early or late hospital readmissions due to asthma (Table 2 and Table 3). Neither was there a correlation between obesity and FEV1 (Table 1). 

## 4. Discussion

The relationship between obesity and asthma has been widely studied, but the relationship between processes continues to be debated due to the lack of agreement between the different studies. 

In our population of asthmatics who required any hospital readmission, more than 71% were obese or overweight, but this is not associated with the incidence of hospital readmissions. Our results are in agreement with the other authors who did not observe a relationship between obesity and exacerbations or with the control of the asthma [8,16,21,29,30,31], although it was different from other studies where we showed a poorer control of asthma or increase in the incidence of the exacerbations with obesity [13,14,15,19,20,32,33,34,35,36].

The disagreement between the results of the various studies, as well as the demonstration that some mechanisms that appear to support a relationship between obesity and asthma, seem to indicate that there probably could be various phenotypes within the asthma-obesity syndrome. In fact, the response to weight loss is very different in patients with early-onset atopic asthma, where the disease does not remit even in the case of weight loss, although it can improve the symptoms. The disease was resolved when they lost weight for those included in the late-onset non-atopic asthma phenotype [37].

Some authors suggest that the discordant results could be due to methodological issues [34]. On the one hand, the BMI may not be a good indicator of body fat since it does not differentiate fat-mass from fat-free mass, being able to be a muscle mass indicator, especially in young males [1]. Furthermore, it is known that the asthma diagnosis in obese patients is more difficult due to over-diagnosis as well as underdiagnosis, as such that it could interfere in drawing precise conclusions from the studies [38]. 

Other studies include patients with an asthma diagnosis based on questionnaires completed by the patients themselves. In these cases, there could be incorrect interpretations of the symptoms, interpreting as asthma that which is due to the obesity [21]. 

It is known that some factors that produce obesity can also lead to a poor progression of the asthma. Thus, animal models demonstrate that a diet rich in fructose leads to systemic metabolic dysfunction, increase in bronchial hyperresponsiveness (BHR), and oxidative stress in the airway [39]. Furthermore, it has been observed in children that a higher consumption of sugary drinks is associated with a higher risk of asthma [40]. Adherence to a Mediterranean diet has also been associated with less obesity and a lower risk of asthma [41,42,43]. These findings suggest that, at least in part, the relationship between obesity and asthma could not be a direct relationship between both, but that it could be determined by other underlying factors. 

Another thing to consider is that some comorbidities are not evaluated in the different studies, which could affect the results obtained. In this sense, it has recently been suggested that there is a possible relationship of asthma with ischaemic heart disease regardless of the presence of other vascular risk factors [44]. Another comorbidity that has been associated with asthma is obstructive sleep apnoea and is not analysed in most studies [45]. Gastroesophageal reflux disease is common in obese patients, and it well known that it is a risk factor for exacerbations of asthma [46].

The possibility of a reverse causation has also been proposed, as such that asthma may be the cause of the obesity, as suggested in other studies, which would complicate the interpretation of the true relationship between obesity and asthma [47,48]. On the other hand, the various pieces of pathophysiological evidence coming from animal models as well as studies in humans seem to support the detrimental effect of the obesity on the asthma prognosis. 

The inflammation of the airway is different between obese and non-obese asthmatics. In a mouse model it was observed that in obese mice, after an ovalbumin challenge, the inflammation in the airway was more intense [49]. Van der Wiel et al. mentioned that in obese patients with mild-moderate asthma there was no correlation between eosinophilia in sputum and that obtained in the submucosa of the airway, presenting with elevated eosinophilia in the submucosa even in the absence of eosinophilia in the sputum. However, in the non-obese there is a correlation between the tissue eosinophilia and the increase of eosinophils in sputum [50].

Hormonal factors could also play a role. The leptin levels increase in obese individuals, and those of adiponectin are reduced [51]. The increase in leptin is higher in obese patients with asthma than in obese ones without asthma [52]. It is known that leptin is a pro-inflammatory adipokine that correlates negatively with lung function and with the increase in reactivity to methacholine, whilst adiponectin has anti-inflammatory properties [52,53,54]. A greater hyperresponsiveness has been observed in cultured human airway smooth muscle cells from obese asthmatics [55].

Another aspect that has been associated with a poor asthma prognosis in obese patients is a lower response to the usual asthma treatments, especially to the corticoids [1,55].

The obesity itself causes a deterioration in lung function, with the functional residual capacity (FRC) and residual volume being particularly affected, as well as leading to a reduction in the FEV1 [56,57,58,59]. The reduction in FRC favours the increase in the BHR, one of the main characteristics of asthma [37]. Even in non-obese individuals, it is known that a greater fat-mass has a negative impact on lung function [60]. Another argument in favour of the relationship between obesity and a poor asthma prognosis is the favourable response to the weight loss, whether by diet and exercise or with bariatric surgery, with improvements in the asthma symptoms and lung function [61,62,63,64].

During the year before the index hospital admission, obese patients had more exacerbations without any difference in hospital admissions. Similarly, they don´t have more frequent readmissions during the follow-up period. In the literature, asthma exacerbations requiring hospital admission are usually a minority, since most patients are discharged from the Emergency Room [14,65].

Finally, our study includes some limitations. On including patients with hospital admissions, they might not be representative of the general asthmatic population. It could be that obese patients are over-represented due to having a higher probability of admissions due to other comorbidities [45,46]. Given that it is a retrospective study, all the variables of all the patients were not available. Furthermore, we didn´t analyse the effect of some relevant characteristics, such as allergic sensitization, because they were available in a few patients. Likewise, all the patients were from a single university hospital, which may affect the external validity of the study. On the other hand, one strength of this study is that the reliability of the asthma diagnosis, when it comes from hospital discharge reports, is elevated [66]. 

In conclusion, obesity does not seem to be a determining factor in the risk of asthma exacerbations. It may have a higher impact in some phenotypes, and other underlying factors, such as the comorbidities common to both diseases, may play a role in this complex interaction. 

## Figures and Tables

**Table 1:** Main characteristics of the included patients.

Variable	Total	Normal Weight	Overweight	Obesity	*p*
Age, mean (SD)	55.6 (19.5)	44.6 (20.7)	58.5 (19.2)	61.5 (14.9)	**0.000**
Gender, *n* (%) Male Female	281 (34.8) 526 (65.2)	87 (37.8) 143 (62.2)	122 (42.4) 166 (57.6)	72 (24.9) 217 (75.1)	**0.000**
FEV1% ≥80% ≥60–80% <60%	307 (38.1) 221 (27.4) 278 (34.5)	98 (42.4) 62 (26.8) 71 (30.7)	105 (36.7) 78 (27.3) 103 (36.0)	104 (36.0) 81 (28.0) 104 (36.0)	0.556
Charlson index 0 1 ≥2	337 (41.7) 266 (32.9) 206 (25.5)	126 (54.5) 78 (33.8) 27 (11.7)	108 (37.5) 98 (34.0) 82 (28.5)	103 (35.5) 90 (31.0) 97 (33.5)	**0.000**
Smokers Never smoker, *n* (%) Current smoker, *n* (%) Former smoker, *n* (%)	380 (59.7) 166 (26.1) 90 (14.2)	89 (46.4) 80 (41.7) 23 (12.0)	139 (62.3) 46 (20.6) 38 (17.0)	152 (68.8) 40 (18.1) 29 (13.1)	**0.000**
Asthma severity Intermittent, *n* (%) Mild, *n* (%) Moderate, *n* (%) Severe, *n* (%)	177 (26.2) 168 (24.9) 162 (24.0) 168 (24.9)	71 (34.6) 53 (25.9) 48 (23.4) 33 (16.1)	64 (27.1) 52 (22.0) 60 (25.4) 60 (25.4)	42 (17.9) 63 (26.9) 54 (23.1) 75 (32.1)	**0.000**
Hospital admissions previous year 0, N (%) 1, N (%) ≥2, N (%)	671 (82.9) 103 (12.7) 35 (4.3)	204 (88.3) 20 (8.7) 7 (3.0)	231 (80.2) 45 (15.6) 12 (4.2)	236 (81.4) 38 (13.1) 16 (5.5)	0.093
Emergency visits previous year 0, N (%) 1–3, N (%) ≥4, N (%)	589 (72.9) 198 (24.5) 21 (2.6)	188 (81.4) 38 (16.5) 5 (2.2)	198 (68.8) 84 (29.2) 6 (2.1)	203 (70.2) 76 (26.3) 10 (3.5)	**0.009**
ICS-LABA at hospital discharge No Yes	244 (30.2) 565 (69.8)	75 (32.5) 156 (67.5)	89 (30.9) 199 (69.1)	80 (27.6) 210 (72.4)	0.456
ICS at hospital discharge No Yes	652 (80.6) 157 (19.4)	184 (79.7) 47 (20.3)	225 (78.1) 63 (21.9)	243 (83.8) 47 (16.2)	0.207
OCS at hospital discharge No Yes	245 (30.3) 564 (69.7)	71 (30.7) 160 (69.3)	99 (34.4) 189 (65.6)	75 (25.9) 215 (74.1)	0.082

SD: standard deviation; n: number of cases; FEV1%: Forced expiratory volume in the first second, percentage of reference value; ICS-LABA: combination inhaled corticosteroids-long acting beta agonists; ICS: inhaled corticosteroids; OCS: oral corticosteroids.

**Table 2:** Number of patients with early and late readmissions according to Body Mass Index using Univariate analysis.

	Early Readmission *n* (%)		*p*	Late Readmission *n* (%)		*p*	Frequent Late Readmission, *n* (%)		*p*	Any Readmission, *n* (%)		*p*
	**0**	**≥1**	**0.37**	**0**	**≥1**	0.44	**0**	**≥2**	0.12	**0**	**≥1**	0.29
**Normal weight,** ***n* (%)**	226 (97.8)	5 (2.2)		189 (81.8)	42 (18.2)		189 (95.5)	9 (4.5)		186 (80.5)	45 (19.5)	
**Overweight,** ***n* (%)**	284 (98.6)	4 (1.4)		228 (79.2)	60 (20.8)		228 (90.5)	24 (9.5)		225 (78.1)	63 (21.9)	
**Obesity,** ***n* (%)**	281 (96.9)	9 (3.1)		224 (77.2)	66 (22.8)		224 (91.4)	21 (8.6)		217 (74.8)	73 (25.2)	

Early readmission: in the following 15 days after hospital discharge (0 versus ≥ 1 readmissions); Late readmission: from 16 days after discharge (0 versus ≥ 1 readmissions); Frequent late readmission: from 16 days after discharge (0 versus ≥ 2 readmissions); *n*: number of cases.

**Table 3:** Multivariate analysis of readmissions according to Body Mass Index.

	OR (CI 95%)
**EARLY READMISSION (0 versus** **≥ 1)**	
Normal weight Overweight Obesity	1 0.410 (0.101-1.668) 0.840 (0.244-2.890)
**LATE READMISSION (0 versus** **≥ 1)**	
Normal weight Overweight Obesity	1 0.734 (0.448-1.203) 0.778 (0.473-1.280)
**FREQUENT LATE READMISSION (0 versus** **≥ 2)**	
Normal weight Overweight Obesity	1 1.351 (0.570-3.204) 1.146 (0.464-2.831)
**ANY READMISSION (0 versus** **≥ 1)**	
Normal weight Overweight Obesity	1 0.858 (0.461-1.597) 0.841 (0.450-1.571)

OR: Odds Ratio; CI: Confidence interval; ICS-LABA: combination inhaled corticosteroids-long acting beta agonists; ICS: inhaled corticosteroids; OCS: oral corticosteroids; Adjusted by age, gender, FEV1% (Forced expiratory volume in the first second), percentage of reference value, Charlson index (comorbidity), smoking habit, asthma severity, frequency of hospital admissions and emergency visits in the previous year, and treatment at hospital discharge (ICS-LABA, ICS, OCS).
